# Supplementary material for: A functional family of fluorescent nucleotide analogues to investigate actin dynamics and energetics
Source: Nat Commun. 2021 Jan 22;12:548. doi: 10.1038/s41467-020-20827-4 (PMC7822861; doi:10.1038/s41467-020-20827-4)
Supplement: Supplementary file 1 — Supplementary Information [file 41467_2020_20827_MOESM1_ESM.pdf]

A functional family of fluorescent nucleotide analogues to investigate actin dynamics and energetics

Jessica Colombo[1], Adrien Antkowiak[1], Konstantin Kogan[2], Tommi Kotila[2], Jenna Elliott[1], Audrey Guillotin[1], Pekka Lappalainen[2] and Alphée Michelot[1]

1 Aix Marseille Univ, CNRS, IBDM, Turing Centre for Living Systems, 13288 Marseille, France
2 HiLIFE Institute of Biotechnology, P.O. Box 56, University of Helsinki, 00014 Helsinki, Finland

Supplementary Information

5 Supplementary Figures
2 Supplementary Tables
1 Supplementary Reference

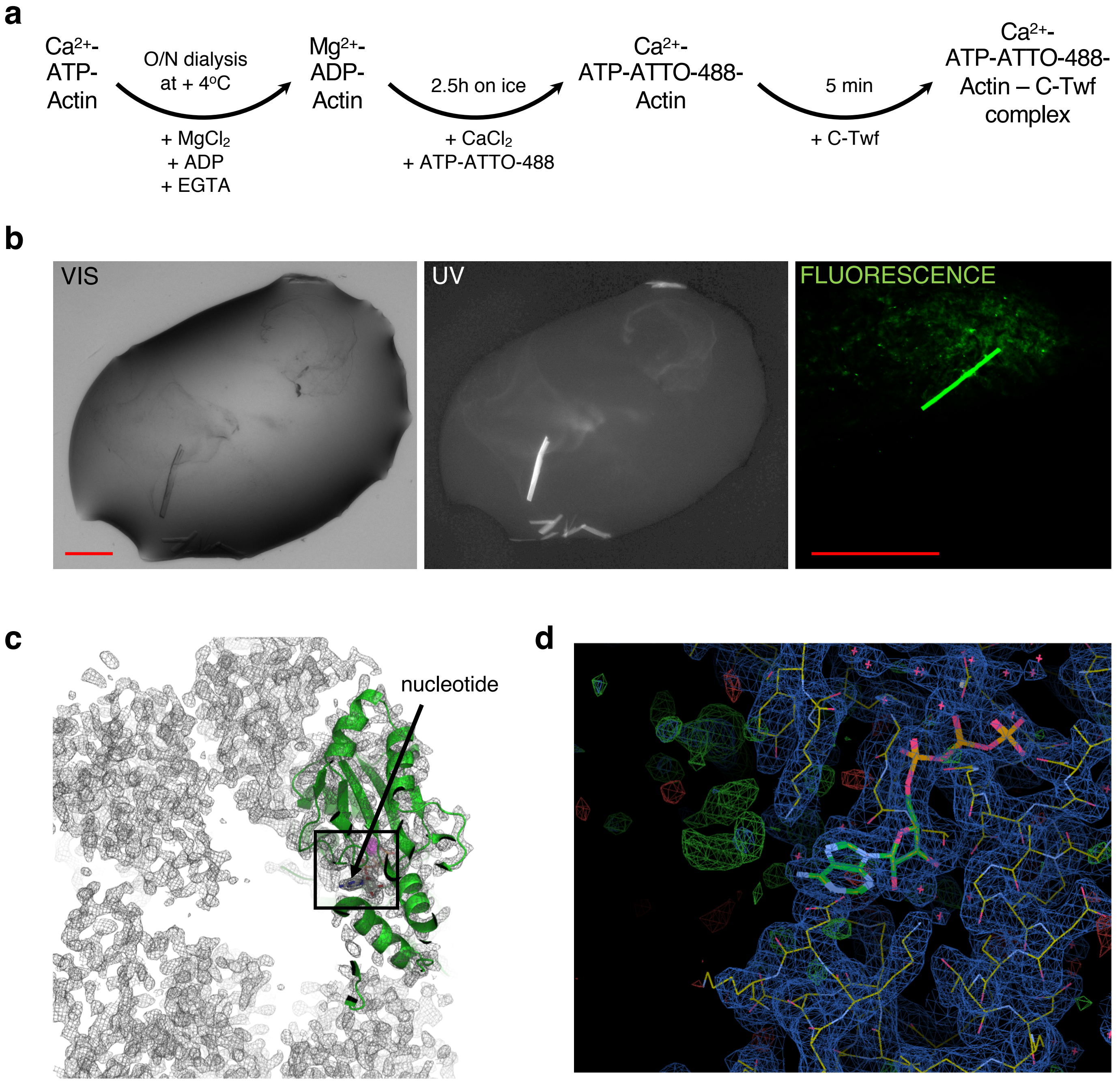

Supplementary Figure 1, related to Figure 1c-f: Crystallization and structural analysis of ATP-ATTO-488 actin. (a) Procedure to ensure efficient exchange of ATP with ATP-ATTO-488 on actin, and complex formation between ATP-ATTO-488 actin with an ADF domain derived from mouse twinfilin-1. Please note that this protein domain inhibits nucleotide exchange, and thus locks the ATP-ATTO-488 in actin 1. (b) Crystals in the drops were visible in white light, UV light, and they emitted fluorescence, indicating the presence of fluorescent nucleotides in protein crystals. Red scale bars: 10 µm. (c) Electron density map (2Fo-Fc at σ = 1.1) of the protein molecules (grey mesh) packed in the crystal. One representative actin molecule is indicated in green ribbon presentation, and the white background represents solvent channels in the crystals that were not occupied by any protein molecule. (d) Structural analysis of ATP-ATTO-488 actin reveals unassigned electron density in both Fo-Fc maps at σ = 2.3 (green) near the nucleotide (bold sticks) and in 2Fo-Fc maps at σ = 1.0 (blue). The extra electron density probably represents partial density of the linker and fluorescent dye, connected to the bound nucleotide (white dash line). In the final deposited maps some of these densities have been modelled with water molecules.

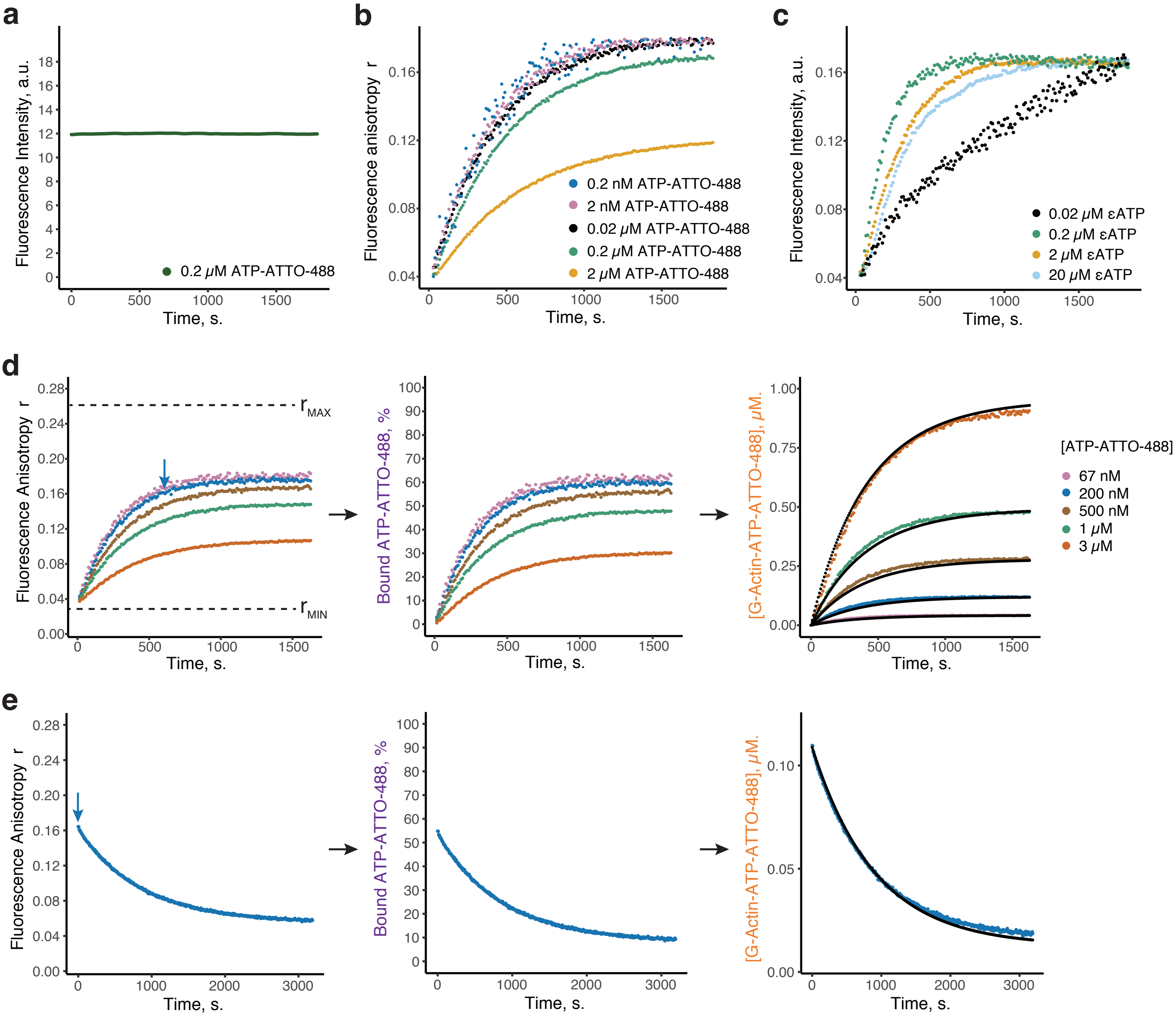

Supplementary Figure 2, related to Figure 2: Binding kinetics of ATP-ATTO-488 and etheno-ATP (εATP) to G-actin (2 μM) in NFG + MEI buffer and detailed methodology for the characterization of the binding kinetics of ATP-ATTO 488 to G-actin. (a) Fluorescence intensity signal of ATP-ATTO-488 (0.2 μM) during nucleotide exchange with G-actin. (b) Binding curves of ATP-ATTO-488 to G-actin showing that the method remains accurate and sensitive for concentrations of ATP-ATTO-488 ranging over several orders of magnitude. Exchange kinetics occur over similar time scales for every concentration of ATP-ATTO-488, although plateaus are lower at high concentration of ATP-ATTO-488 since a higher fraction of ATP-ATTO-488 remains unbound. (c) Binding curves of εATP to G-actin showing that in similar conditions, εATP is not sensitive enough to record binding kinetics below approximately 0.2 μM. (d) Binding kinetics (colored data) and fit curves (black) in the presence G-actin and increasing concentration of ATP-ATTO 488. Left graph shows fluorescence anisotropy data (Please note that curves reach different plateau values because the fraction of bound ATP-ATTO-488 to G-actin decreases at steady-state when the total concentration of ATP-ATTO-488 increases), from which percentage of bound ATP-ATTO-488 is deduced in the middle graph (measured r_{MIN} = 0.036 and r_{MAX} = 0.27), and from which concentration of G-actin-ATP-ATTO-488 is calculated in the right graph. (e) Kinetics (blue data from the 200 nM ATP-ATTO-488 condition of (d)) and fit curve (black) of ATP-ATTO-488 dissociation from G-actin in the presence of an excess of ATP (100 μM). Left, middle and right graphs plot anisotropy data, percentage of bound ATP-ATTO-488 and concentration of G-actin-ATP-ATTO-488 as in (d). Source data are provided as a Source Data file.

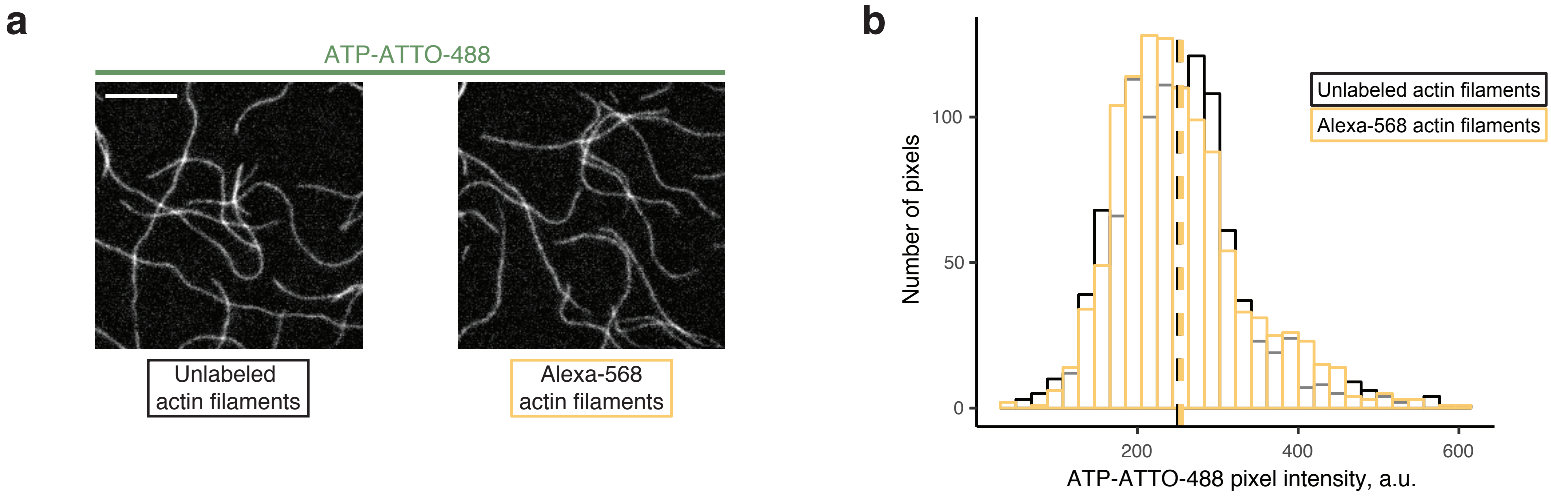

Supplementary Figure 3, related to Figure 3b: Fluorescence of ATP-ATTO-488 bound to filaments assembled from unlabled actin monomers or Alexa-568-labeled (10%) actin monomers. (a) 10 µM ATP-ATTO-488 was pre-incubated with 10 µM actin (unlabeled or 10% Alexa568-labeled) for 2 h at room temperature in NFG buffer to reach 39% occupancy. Polymerization was then induced between slides and coverslips by 10-fold dilution in imaging buffer 2 supplemented with 3 µM profilin. Images of actin filaments were taken 10 min later. This experiment was repeated independently three times. Scale bar: 10 µm. (b) Distribution of ATP-ATTO-488 pixel intensities along actin filaments in (a), with mean values of 251.3 a.u. (n=1077) for filaments assembled from unlabled actin (black) and 254.3 a.u. (n=1116) for filaments assembled from Alexa-568-labeled actin (yellow) (One-factor ANOVA test p value = 0.37). Source data are provided as a Source Data file.

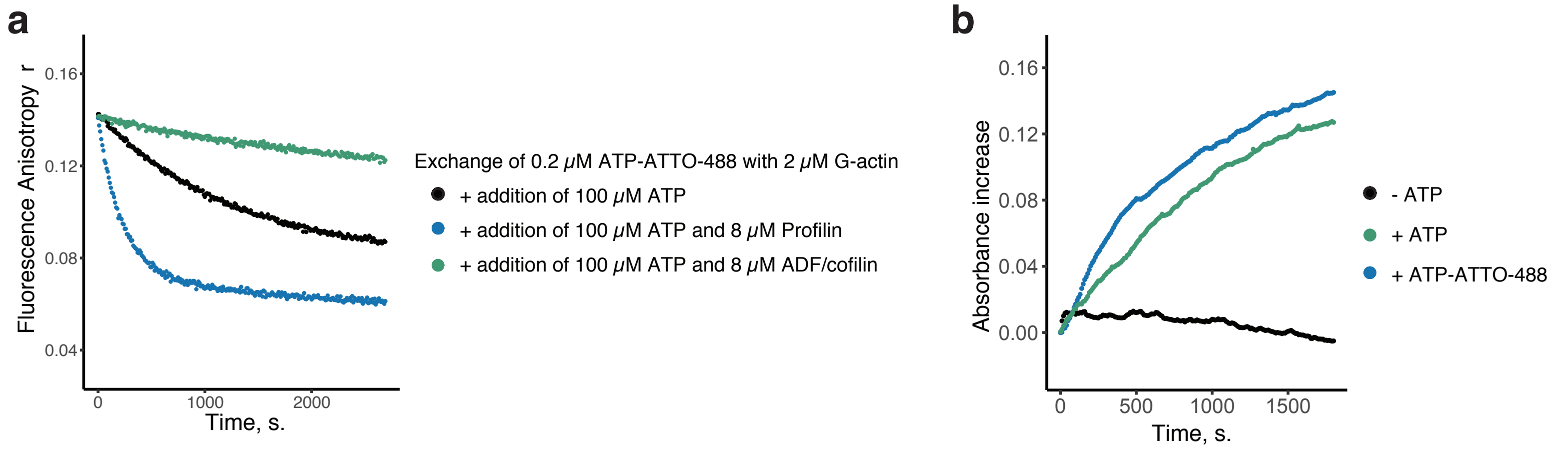

Supplementary Figure 4, related to Figure 4a, d and i: Additional data on the use of ATP-ATTO-488 with actin and ABPs. (a) Kinetics of ATP-ATTO-488 dissociation from G-actin in the presence of an excess of ATP (100 µM) and in the absence (black) or in the presence of 8 µM profilin (blue) or 8 µM ADF/cofilin (green). Actin monomers (2 µM) were exchanged with ATP-ATTO-488 (0.2 µM) for 30 min at room temperature in NFG + MEI buffer prior to addition of ATP and profilin or ADF/cofilin. Analysis of slopes at initial time-points indicate 4-fold and 0.2-fold increase of nucleotide exchange rates by profilin and ADF/cofilin, respectively. (b) Time-course of phosphate release from actin filaments measured in the presence of F-actin (25 µM), profilin (1.5 µM), ADF/cofilin (1.5 µM), MESG (0.2 mM), PNP (2 units) and in the absence (black) or in the presence of ATP (33 µM; green) or ATP-ATTO-488 (33 µM; blue). Source data are provided as a Source Data file.

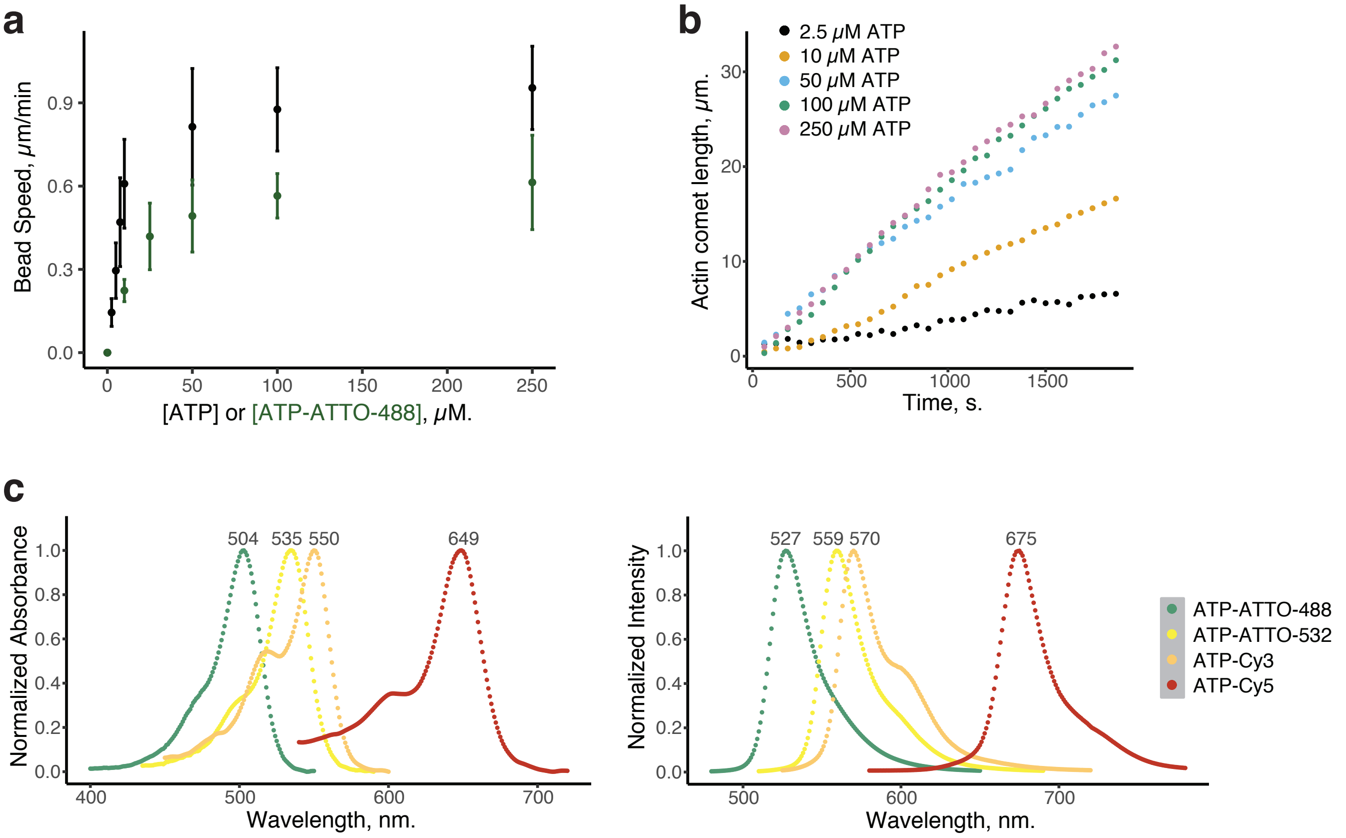

Supplementary Figure 5, related to Figure 5: Precise characterization of the potency of fluorescent ATPs as energy sources for actin-based force generation. (a) A quantification of the experiment presented in Figure 5b. Average bead velocity for various concentrations of unlabeled ATP or ATP-ATTO-488. For conditions with ATP, n = 13, 23, 27, 32, 28, 25 and 23 at 2.5 nM, 5 nM, 7.5 nM, 10 nM, 50 nM, 100 nM and 250 nM, respectively. For conditions with ATP-ATTO-488, n = 23, 21, 17, 13 and 16 at 10 nM, 25 nM, 50 nM, 100 nM and 250 nM, respectively. Data are presented with mean values and standard deviations. (b) Another quantification from the experiment presented in Figure 6b. Representative examples of comet length changes over time for various concentration of unlabeled ATP. (c) Absorbance (left) and emission spectra (right) of four fluorescent ATPs identified in this study that are functional for actin-based force generation. Source data are provided as a Source Data file.

Supplementary Table 1: **Data collection and refinement statistics (molecular replacement)**

ATP-ATTO488-Actin	
Data collection	
Space group	P212121
Cell dimensions	
a, b, c (Å)	50.55, 69.02, 147.95
α, β, γ (°)	90.00, 90.00, 90.00
Resolution (Å)	62.55-2.56 (2.61-2.56)*
Total number of reflections	102 863 (5042)
Number of unique reflections	17 247 (822)
R_{merge}	0.146 (0.885)
R_{meas}	0.161 (0.969)
R_{pim}	0.090 (0.525)
$I / \sigma I$	10.3 (2.3)
Completeness (%)	99.8 (100.0)
Multiplicity	6.0 (6.1)
$CC_{1/2}$	0.996 (0.701)
Refinement	
Resolution (Å)	62.55-2.564
No. reflections	17247
R_{work} / R_{free}	0.17/0.23 (0.22/0.33)
No. atoms	
Protein	3994
Ligand/ion	32
Water	245
B-factors	
Wilson B	38.87
Mean B	42.01
Protein	41.9
Ligand/ion	32.6
Water	45.7
R.m.s. deviations	
Bond lengths (Å)	0.013
Bond angles (°)	1.7
Ramachandran plot	
Favored (%)	98
Allowed (%)	1.8
Outliers (%)	0.2
Molprobity	
Overall score	1.65
Clash score (all atoms)	3.88

* Values in parentheses are for highest-resolution shell.

Supplementary Table 2 : Summary of affinities and rate constants measured in this study

Chemical reaction	Buffer conditions	k_+	k_-	K_d
G + ATP = G-ATP	ME buffer	12 $\mu M^{-1}.s^{-1}$ (*)	(2.8 +/- 0.3) x 10^{-3} s^{-1} (**)	0.24 nM (*)
G + ATP-ATTO-488 = G-ATP-ATTO-488	ME buffer	10 $\mu M^{-1}.s^{-1}$ (*)	(1 +/- 0.4) x 10^{-3} s^{-1} (**)	0.1 nM (*)
G-ATP + Profilin = G-ATP-Profilin	G-buffer			2.1 +/- 0.5 µM
G-ATP-488 + Profilin = G-ATP-488-Profilin	G-buffer			2.6 +/- 0.6 µM
G-ATP + ADF/cofilin = G-ATP-ADF/cofilin	G-buffer			1.7 +/- 0.5 µM
G-ATP-ATTO-488 + ADF/cofilin = G-ATP-ATTO-488-ADF/cofilin	G-buffer			1.6 +/- 0.3 µM

(*) non-sensitive parameters used in the model to fit our data
(**) sensitive parameters used in the model to fit our data

Supplementary Reference

1. Ojala, P. J. et al. The Two ADF-H Domains of Twinfilin Play Functionally Distinct Roles in Interactions with Actin Monomers. Mol. Biol. Cell 13, 3811–3821 (2002).
