# Supplementary material for: A functional family of fluorescent nucleotide analogues to investigate actin dynamics and energetics
Source: Nat Commun. 2021 Jan 22;12:548. doi: 10.1038/s41467-020-20827-4 (PMC7822861; doi:10.1038/s41467-020-20827-4)
Supplement: Supplementary file 3 — Description of Additional Supplementary Files [file 41467_2020_20827_MOESM3_ESM.pdf]

Description of Additional Supplementary Files

File name: Supplementary Movie 1
Description: Time lapse TIRFM-movie of a simultaneous exchange of ATP-ATTO-488 (0.25 µM) and polymerization of actin (0.4 µM; 7.5 % Alexa 568-labeled) in the presence of profilin (0.8 µM). Total elapsed time of 30 min was compressed to a 9-second movie.

File name: Supplementary Movie 2
Description: Time lapse TIRFM-movie of an actin filament labeled with ATP-ATTO-488. Photobleaching of ATP-ATTO-488 shows absence of fluorescence recovery. Total elapsed time of 15 min was compressed to a 11-second movie.
