# Supplementary material for: A functional family of fluorescent nucleotide analogues to investigate actin dynamics and energetics
Source: Nat Commun. 2021 Jan 22;12:548. doi: 10.1038/s41467-020-20827-4 (PMC7822861; doi:10.1038/s41467-020-20827-4)
Supplement: Supplementary file 6 — Reporting Summary [file 41467_2020_20827_MOESM6_ESM.pdf]

nature research

Corresponding author(s): Dr. Alphée Michelot

Last updated by author(s): Dec 10, 2020

Reporting Summary

Nature Research wishes to improve the reproducibility of the work that we publish. This form provides structure for consistency and transparency in reporting. For further information on Nature Research policies, see our Editorial Policies and the Editorial Policy Checklist.

Statistics

For all statistical analyses, confirm that the following items are present in the figure legend, table legend, main text, or Methods section.

n/a	Confirmed	
[ ]	[x]	The exact sample size (*n*) for each experimental group/condition, given as a discrete number and unit of measurement
[ ]	[x]	A statement on whether measurements were taken from distinct samples or whether the same sample was measured repeatedly
[ ]	[x]	The statistical test(s) used AND whether they are one- or two-sided<br>*Only common tests should be described solely by name; describe more complex techniques in the Methods section.*
[x]	[ ]	A description of all covariates tested
[x]	[ ]	A description of any assumptions or corrections, such as tests of normality and adjustment for multiple comparisons
[ ]	[x]	A full description of the statistical parameters including central tendency (e.g. means) or other basic estimates (e.g. regression coefficient) AND variation (e.g. standard deviation) or associated estimates of uncertainty (e.g. confidence intervals)
[ ]	[x]	For null hypothesis testing, the test statistic (e.g. *F*, *t*, *r*) with confidence intervals, effect sizes, degrees of freedom and *P* value noted<br>*Give P values as exact values whenever suitable.*
[x]	[ ]	For Bayesian analysis, information on the choice of priors and Markov chain Monte Carlo settings
[x]	[ ]	For hierarchical and complex designs, identification of the appropriate level for tests and full reporting of outcomes
[ ]	[x]	Estimates of effect sizes (e.g. Cohen's *d*, Pearson's *r*), indicating how they were calculated

Our web collection on statistics for biologists contains articles on many of the points above.

Software and code

Policy information about availability of computer code

Data collection	Standard fluorescence imaging: Zen 2.3 blue edition; TIRFM imaging: Metamorph v.7.7.10.0; Spectrofluorimetry: Safas SP2000 v.7.8.13.0; X-ray detection: XDS program package Mar 15, 2019 (BUILT 20191015)
Data analysis	Fluorescence microscopy image analysis: ImageJ v.10.2; Spectrofluorimetry curve fit: KinteK Explorer v.1; X-Ray pattern analysis: AIMLESS (CCP4 v. 7.0.078) utilizing autoPROC toolbox v. 1.0.5; Molecular replacement: Phaser-MR from the Phenix package (version 1.18.2) and refinement with BUSTER v.2.10.3; Other structure analysis tools: , POINTLESS v. 1.11.21 and STARANISO v. 2.3.13; Other data analysis: R v.3.4.3, including package ggplot2 version 2.2.1 for data representation, and Microsoft Excel for Mac v.16.16.13

For manuscripts utilizing custom algorithms or software that are central to the research but not yet described in published literature, software must be made available to editors and reviewers. We strongly encourage code deposition in a community repository (e.g. GitHub). See the Nature Research guidelines for submitting code & software for further information.

Data

Policy information about availability of data

All manuscripts must include a data availability statement. This statement should provide the following information, where applicable:

- Accession codes, unique identifiers, or web links for publicly available datasets
- A list of figures that have associated raw data
- A description of any restrictions on data availability

The data that support the findings of this study are available in the article, including Supplementary Files, or are available from the authors upon reasonable request

Field-specific reporting

Please select the one below that is the best fit for your research. If you are not sure, read the appropriate sections before making your selection.

- [x] Life sciences
- [ ] Behavioural & social sciences
- [ ] Ecological, evolutionary & environmental sciences

For a reference copy of the document with all sections, see nature.com/documents/nr-reporting-summary-flat.pdf

Life sciences study design

All studies must disclose on these points even when the disclosure is negative.

Sample size	All experiments were carried out in vitro. For steady-state fluorescence anisotropy experiments, measurements at our spectrofluorimeter indicated that average and standard deviation values do not change significantly above n=10-20 measurments (depending on ATP-ATTO-488 concentration). For this reason, we systematically measured n>20 anisotropy values for each conditions. For phosphate release experiments, we were limited by the large quantity of ATP-ATTO-488 required for this experiment. We measured statistically relevant differences after analyzing n = 5 samples and presented all the results. For microscopy experiments, we analyzed increasing number of actin filaments or beads until we obtained statistically significant results.
Data exclusions	No data were excluded from the analyses
Replication	All experiments presented in this manuscript were performed independently at least three times. All attempts at replication were successful. We present results from one set of experiments, so that results do not include additional statistical variability that might arise from the repetition of the experiments.
Randomization	Actin filaments and comet tails were randomly selected among many for analysis.
Blinding	Not relevant to in vitro work. Data from different experiments were analyzed with the exact same protocoles so that no bias would be introduced.

Reporting for specific materials, systems and methods

We require information from authors about some types of materials, experimental systems and methods used in many studies. Here, indicate whether each material, system or method listed is relevant to your study. If you are not sure if a list item applies to your research, read the appropriate section before selecting a response.

Materials & experimental systems

n/a	Involved in the study	
[x]	[ ]	Antibodies
[x]	[ ]	Eukaryotic cell lines
[x]	[ ]	Palaeontology and archaeology
[x]	[ ]	Animals and other organisms
[x]	[ ]	Human research participants
[x]	[ ]	Clinical data
[x]	[ ]	Dual use research of concern

Methods

n/a	Involved in the study	
[x]	[ ]	ChIP-seq
[x]	[ ]	Flow cytometry
[x]	[ ]	MRI-based neuroimaging
